# Physical activity and risk of Metabolic Syndrome in an urban Mexican cohort

**DOI:** 10.1186/1471-2458-9-276

**Published:** 2009-07-31

**Authors:** Pablo Méndez-Hernández, Yvonne Flores, Carole Siani, Michel Lamure, L Darina Dosamantes-Carrasco, Elizabeth Halley-Castillo, Gerardo Huitrón, Juan O Talavera, Katia Gallegos-Carrillo, Jorge Salmerón

**Affiliations:** 1 Equipe MA2D – Laboratoire ERIC – Université Lyon 1 et Lyon 2, France; 2 Facultad de Ciencias de la Salud, Universidad Autónoma de Tlaxcala, México; 3 Unidad de Investigación Epidemiológica y en Servicios de Salud, Instituto Mexicano del Seguro Social, delegación Morelos, México; 4 UCLA Department of Health Services, School of Public Health and Jonsson Comprehensive Cancer Center, Los Angeles, California, USA; 5 Unidad de Enseñanza, Investigación y Calidad del Instituto de Salud de Estado de México, Toluca, México; 6 Centro de Investigación en Ciencias Médicas, Universidad Autónoma del Estado de México, Toluca, México; 7 Unidad de Investigación Médica en Epidemiología Clínica del Hospital de Especialidades, Centro Médico Nacional Siglo XXI, Instituto Mexicano del Seguro Social, Distrito Federal, México

## Abstract

**Background:**

In the Mexican population metabolic syndrome (MS) is highly prevalent. It is well documented that regular physical activity (PA) prevents coronary diseases, type 2 diabetes and MS. Most studies of PA have focused on moderate-vigorous leisure-time activity, because it involves higher energy expenditures, increase physical fitness, and decrease the risk of MS. However, for most people it is difficult to get a significant amount of PA from only moderately-vigorous leisure activity, so workplace activity may be an option for working populations, because, although may not be as vigorous in terms of cardio-respiratory efforts, it comprises a considerable proportion of the total daily activity with important energy expenditure. Since studies have also documented that different types and intensity of daily PA, including low-intensity, seem to confer important health benefits such as prevent MS, we sought to assess the impact of different amounts of leisure-time and workplace activities, including low-intensity level on MS prevention, in a sample of urban Mexican adults.

**Methods:**

The study population consisted of 5118 employees and their relatives, aged 20 to 70 years, who were enrolled in the baseline evaluation of a cohort study. MS was assessed according to the criteria of the National Cholesterol Education Program, ATP III and physical activity with a validated self-administered questionnaire. Associations between physical activity and MS risk were assessed with multivariate logistic regression models.

**Results:**

The prevalence of the components of MS in the study population were: high glucose levels 14.2%, high triglycerides 40.9%, high blood pressure 20.4%, greater than healthful waist circumference 43.2% and low-high density lipoprotein 76.9%. The prevalence of MS was 24.4%; 25.3% in men and 21.8% in women. MS risk was reduced among men (OR 0.72; 95%CI 0.57–0.95) and women (OR 0.78; 95%CI 0.64–0.94) who reported an amount of ≥30 minutes/day of leisure-time activity, and among women who reported an amount of ≥3 hours/day of workplace activity (OR 0.75; 95%CI 0.59–0.96).

**Conclusion:**

Our results indicate that both leisure-time and workplace activity at different intensity levels, including low-intensity significantly reduce the risk of MS. This finding highlights the need for more recommendations regarding the specific amount and intensity of leisure-time and workplace activity needed to prevent MS.

## Background

Metabolic Syndrome (MS) is a common disorder caused by a combination of unhealthy diet, sedentary lifestyle and genetic predisposition [1]. This syndrome is a major risk factor for several chronic diseases, mainly type 2 diabetes and cardiovascular diseases [2-5]. Some components of MS are highly prevalent in the Mexican population. These include high rates of central obesity [6], low levels of high density lipoprotein cholesterol (HDL), hypertriglyceridemia, hypoalphalipoproteinemia [7] and type 2 diabetes [8]. In fact, cardiovascular disease is the leading cause of death for both sexes, and type 2 diabetes is the second cause of death among women, and the third among men in Mexico [9].

Epidemiological studies have demonstrated that moderate-vigorous daily physical activity (PA) prevents both the incidence of chronic diseases and premature death [10]. It is also well documented that habitual leisure-time (LT) activity prevents elevated blood pressure, insulin resistance, glucose intolerance [11], elevated triglycerides, low levels of HDL and decreases body weight [12], preventing the development of coronary heart diseases [13], type 2 diabetes and MS [14-17].

Public health recommendations regarding the type and amounts of PA needed to improve and maintain health benefits among adults have been established in the USA [18]. In Mexico, the National Commission of Sport and the Mexican Ministry of Health (MMH) have adopted the USA recommendations: people should engage in moderate intensity PA at least five days a week for 30 minutes, or vigorous activity at least three days a week for 20 minutes [19,20]. These recommendations represent an adequate amount of PA for general health promotion and disease prevention [21], but the type, amount and intensity of PA required to prevent or reverse MS has not been well established [22,23].

Most studies of PA have focused on moderate-vigorous LT activity, because it involves higher energy expenditures [24], increase physical fitness [25], and has demonstrated to decrease the risk of MS [14-17]. However, for most people it is difficult to get a significant amount of PA from moderate-vigorous LT activity, so other types of PA that can improve health should also be explored [26]. For example, workplace (WP) activity may be an option for working populations, because, although WP activities may not be as vigorous in terms of cardio-respiratory efforts, they do make up a considerable proportion of the total daily PA [27,28] with an important energy expenditure [22,29].

Prospective studies have shown that in order to prevent MS, energy expenditure seems to be more important than intensity, independently of aerobic fitness and obesity, [22,30,31]. Since, studies have also documented that different types and intensity of daily PA, including low-intensity, seem to confer important health benefits such as decreased blood glucose concentrations [31], lower risk of type 2 diabetes [32], and helping to prevent MS [22]; the aim of this study is to assess the impact of different amounts of LT and WP activities, including low-intensity level on MS prevention, in a sample of urban Mexican adults.

## Methods

### Study population

The study population consisted of adult participants in the Health Worker Cohort Study (HWCS), including workers and their relatives from the "Instituto Mexicano del Seguro Social" (IMSS), the "Instituto Nacional de Salud Pública" (INSP) and from the "Universidad Autónoma del Estado de México" (UAEM). This ongoing cohort study is focusing on the relationship between certain lifestyle factors and health. From March 2004 to April 2006, 8315 adults were formally enrolled in the cohort study. The specifics of the study design, methodology and participants' baseline characteristics have been detailed elsewhere [33-35]. The ethics committees of all participating institutions approved the study protocol and informed consent forms.

For this study we performed a cross-sectional analysis of data from a sample of 7991 adults participating in the HWCS, aged 20 to 70 years. To avoid any health conditions that could interfere with the participants' ability to engage in PA [36], we excluded: 1) participants diagnosed any component of MS, such as type 2 diabetes (n = 174), hypertension (n = 581), or any type of dyslipidemia (n = 619); and 2) those with a serious chronic disease, such as cardiovascular disease (n = 41), cerebrovascular disease (n = 8), any type of cancer (n = 72), cirrhosis (n = 6), pulmonary diseases (n = 192), kidney failure (n = 18), rheumatoid or degenerative arthritis (n = 270), hip or femur fracture (n = 26), Parkinson's disease (n = 4) and depression (n = 170), or a combination of these diseases (n = 692). Our sample consisted of the remaining 5118 participants. These diseases were identified based on the participants' responses in the self-administered questionnaire (from questions about hospitalizations and medication prescriptions), as well as the results of blood tests, clinical and anthropometrical examinations.

### Physical activity assessment

Physical activity was assessed using a validated physical activity questionnaire version 2002, used in the "Health Professionals Follow-up Study" [37,38], validated in Spanish [39], and adapted for Mexican urban population as follows: 1) adding some LT activities that are common among the Mexican population such as football and fronton, as well eliminating weightlifting and sailing, which are less common, and 2) adding more specific choices to the WP domain such as carrying moderate or heavy loads, pushing objects, climbing the stairs and using tools.

Participants were asked to report the amount of time they spent engaged in specific activities during their LT and WP contexts. The PA section on LT activity included 16 items on the amounts of weekly time spent performing exercises like walking, running, cycling, etc. To obtain the LT activity expended daily, time and frequency spent on each activity were added and the total was divided by 7. To compare the impact of LT activity on risk of MS, we defined two categories: <30 minutes per day and ≥30 minutes per day, based on the minimum time recommended in the PA guidelines for Mexican adults [19,20].

Workplace activity was evaluated using 8 items that address the daily time spent performing work-related activities such as sitting down, standing up, walking, walk lifting objects, and using heavy machinery. To obtain the daily WP activity, we added the duration of these different activities. We defined two WP activity categories based on the median hours of WP activity per day: <3 hours and ≥3.

### Metabolic equivalents assessment

In order to make our results more comparables with other studies, we computed the median of the Metabolic Equivalents (METs) and the kilocalorie equivalency for the time spent in the LT and WP categories, using the updated compendium of physical activity [24]. One MET is the metabolic energy expended by lying quietly and is equivalent to 1 kcal per kg per hour. For instance, a 70 kg person walking at a moderate pace (MET value of 3.5) for 1 hour expends 3.5 METs or 245 kcal [12].

### Clinical and anthropometric evaluation

Waist circumference was measured with a steel measuring tape at the high point of the iliac crest at the end of normal expiration, to the nearest 0.1 cm. Weight was assessed on participants wearing minimal clothing with a previously calibrated electronic TANITA scale. Height was measured with a conventional stadiometer. Body mass index (BMI) was calculated as a ratio of weight (Kg) to height squared (m^2^).

Blood pressure was measured with an electronic digital blood pressure monitor. Participants were seated with their right arm resting at heart level. For the participants from the UAEM three blood pressure measurements were obtained and the mean of the last two measurements was used. For the participants from the INSP and the IMSS, one blood pressure measurement was obtained. Measurement of these anthropometric criteria and blood pressure were performed by nurses trained to perform standardized procedures (reproducibility was evaluated, resulting in a concordance coefficient of 0.83–0.90).

Fasting venous blood samples were collected. Glucose levels were assessed with the oxidize glucose method. Triglycerides were determined with a colorimetric method after enzymatic hydrolysis with lipases technique, and HDL cholesterol by the elimination of chylomicron and subsequent catalase. All biomedical essays were performed at the IMSS laboratory in Cuernavaca, and at the UAEM in Toluca. Both laboratories used procedures standardized according to the proceedings of the International Federation of Clinical Chemistry and Laboratory Medicine [40].

### Metabolic syndrome assessment

The National Cholesterol Education Program, Adult Treatment Panel III (NCEP ATP III) defined MS as the presence of three or more of the following five components: plasma glucose ≥110 mg/dL but <126 mg/dL (≥6.09 but <6.99 mmol/L), serum triglycerides ≥150 mg/dL (≥1.7 mmol/L), systolic and/or diastolic blood pressure: ≥130, but <140 and/or ≥85, but <90 mmHg, respectively; waist circumference ≥102 cm (40 in) for men and ≥88 cm (35 in) for women, and low HDL cholesterol <50 and <40 mg/dL (<1.295 mmol/L and <1.036 mmol/L) for men and women, respectively [41]. We used these parameters in our analysis, although we lowered the cut-point for healthy plasma glucose to ≥100 mg/dL and <126 mg/dL, to optimize our ability to assess diabetes risk [42].

### Demographic characteristics, education, smoking assessment and diet

Demographic data, including level of education, were obtained via the self-applied questionnaire. Smoking status was assessed using the categorization proposed by the World Health Organization: current, past and never [43].

A semi-quantitative Food Frequency Questionnaire (FFQ) validated in the Mexican population [44] was used to assess diet. This questionnaire includes data on the frequency of consumption of 116 foodstuffs during the previous year. Participants' calorie, saturated fat, and alcohol consumption were estimated using a FFQ for the past year. Alcohol consumption was categorized as: non-drinkers (people who have not consumed any alcohol in the last 12 months), moderate drinkers (<2 drinks a day for men, and <1 drink a day for women), and heavy drinkers (≥2 drinks a day for men and ≥1 drink a day for women) [45].

### Analysis

Extreme outliers in the LT and WP measures were identified and removed using the generalized extreme studentized deviation many-outlier method [46]. Differences between the median of METs expenditure in LT and WP categories were computed and tested, using Pearson chi-squared test. Differences between the crude and adjusted prevalences of MS and their components across LT and WP activities were evaluated and tested by sex, using the likelihood test for difference of two probabilities. To estimate the magnitude of the association between MS and daily physical activities, adjusted odds ratios and 95% confidential intervals (CIs) were computed using unconditional and multivariate logistic regression. Adjusted prevalences and adjusted odds ratios were computed by sex. The covariates used to adjust the prevalences and odds ratios were: age, calorie intake, alcohol consumption, education and smoking. Models were also adjusted by LT and WP activities. Analyses were performed using stata version 9.1 (stata Corporation, College Station, Texas, USA).

## Results

The study population was composed of mainly middle-aged participants, more than seventy percent were women, and most participants had elementary and secondary education. Close to 60 percent had a BMI ≥25 Kg/m^2^. Nearly 80 percent reported spending <30 minutes/day in LT activities, and nearly 55% indicated that they spend <3 hours/day engaged in WP activities. Other demographic and lifestyle characteristics of the study population are shown in Table 1.

**Table 1:** Demographic and lifestyle characteristics of study participants (n = 5118).

Age, mean years (SD)	38 (± 11.7)
Sex (%)	
Men	29.0
Women	71.0
Education (%)	
Elementary/secondary education	40.2
High school	19.7
Bachelor's degree or higher	40.1
Occupation (%)	
Administrative staff	33.9
Technicians and cleaning staff	3.4
Health employees and students	14.0
Professors and researchers	13.1
Housewives, retires and unemployed	22.5
Others	13.1
Body Mass Index (%)	
Normal (< 25 kg/m^2^)	44.2
Overweight (25–29 kg/m^2^)	40.6
Obese (> 30 kg/m^2^)	15.2
Mean, kg/m^2 ^(SD)	26 (± 4.5)
Physical activity (%)	
Leisure-time (< 30 min/day)	78.2
Workplace (< 3 hours/day)	54.5
Smoking status (%)	
Current	17.9
Past	23.8
Alcohol intake (%)	
Moderate drinkers	49.9
Heavy drinkers	3.8

The prevalence of MS in the study population was 24.4%. The prevalence of MS among participants with a normal BMI was 6.5% (95% CI 5.5–7.6), among overweight participants it was 28.5% (95% CI 27.0–29.7), and 57.1% (95% CI 53.5–60.8) among obese participants. The prevalence of each MS component was: high blood glucose 14.2%, high triglycerides 40.9%, high blood pressure 20.4%, higher-than-recommended waist circumference 43.2% and low HDL 76.9% (data not shown).

**Figure 1:**
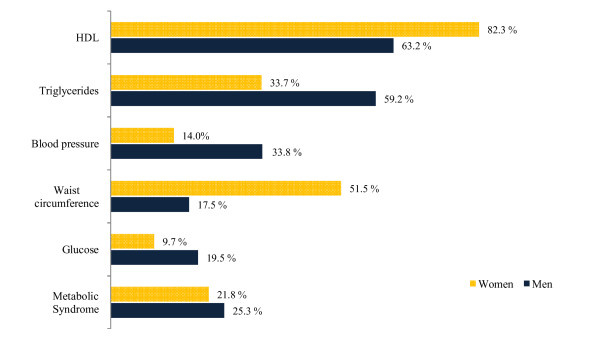
**Prevalence of metabolic syndrome and its components by sex (n = 5118)**. Probability of chi-squared test: p < 0.000 in all cases.

Figure 1 shows the prevalence of MS and each MS component by sex. Low HDL and large waist circumference were the most prevalent MS components among women, and high triglycerides, high blood pressure and high blood glucose were more prevalent among men (p < 0.000, in all cases). Moreover, the prevalence of MS was higher among men than women (25.3 vs 21.8, respectively; p < 0.000).

Table 2, shows the median METs expended per day in each category of LT and WP activities. Participants who engaged in ≥30 minutes of LT activity a day had a higher median METs expenditure than those who reported <30 minutes of LT activity per day (5.3 METs vs. 0.25 METs, respectively; p < 0.000). Participants who reported ≥3 hours of WP activity per day showed higher median of METs expenditure than those who reported <3 hours of WP activity per day (10.8 vs. 1.6 METs, respectively; p < 0.000).

**Table 2:** Median of METs and kilocalories spent in leisure-time and workplace activities.

Leisure-time activity	Workplace activity
(Median*, SD)	(Median*, SD)
	
< 30 minutes per day (n = 4001)	< 3 hours per day (n = 2025)
METs and kilocalorie expenditure in this category:	METs and kilocalorie expenditure in this category:
METs: 0.25 SD (0.54)	METs: 1.6 SD (2.4)
Kcal: 16.1 SD (34.8)	Kcal: 103.2 SD (154)
Example of time activity for METs expenditure in this category:	Examples of time activity for METs expenditure in this category:
Walking at slow pace (< 30 min/mile), during ≈7.5 min (MET value: 2.0 METs/hour).	Sitting meetings, talking, business meeting, during ≈64 min (MET value: 1.5 METs/hour).
	
≥ 30 minutes per day (n = 1117)	≥ 3 hours per day (n = 1691)
METs and kilocalorie expenditure in this category:	METs and kilocalorie expenditure in this category:
METs: 5.3 SD (5.8)	METs: 10.8 SD (3.2)
Kcal: 341.8 SD (347)	Kcal: 696.6 SD (206)
Examples of time activity for METs expenditure in this category:	Examples of time activity for METs expenditure in this category:
Running 10 min/mile, bicycling 4 min/mile or swimming vigorous effort, during ≈34 min (MET value: 10.0 METs/hour).	Walking slowly at 2.5 mph, carrying light objects, or patient care during ≈216 min (MET value: 3.0 METs/hour).

*Pearson chi-squared test for differences between the median of METs expenditure in LT categories and WP categories: p < 0.000 in all cases.

1 mile = 1.61 Kilometers

The prevalence of MS and its components for men and women, by LT and WP activities, are shown in Table 3. The adjusted prevalence of MS and each component of MS were lower among men and women who reported at least 30 minutes per day of LT activity, than in those who spent less than 30 minutes per day. Men who met the physical activity recommendations also showed a significantly lower prevalence of abnormal HDL, large waist circumference and MS, than those who did not. Women who spent at least 30 minutes per day of LT activity showed a significantly lower prevalence of abnormal triglycerides, large waist circumference and MS, than in those who spent less than 30 minutes per day engaged in LT activities.

**Table 3:** Adjusted prevalence of metabolic syndrome and its components by sex, according to leisure-time and workplace activities

Metabolic syndrome and its components	Leisure-time (n = 5118)			Workplace (n = 3716)		
		
	<30 min/day	≥30 min/day	p value	<3 hours/day	≥3 hours/day	p value
	n = 4001	n = 1117		n = 2025	n = 1691	
	
Glucose ≥100 mg/dl						
Men (%)	19.4	18.3	0.530	21.1	18.7	0.499
95% CI	(17.1–22.8)	(15.2–22.1)		(15.1–28.6)	(16.6–21.1)	
Women (%)	10.5	9.6	0.467	13.3	9.6	0.048
95% CI	(9.3–11.8)	(7.8–11.8)		(9.9–15.2)	(8.5–10.8)	
		
Triglycerides ≥ 150 mg/dl						
Men (%)	58.3	53.5	0.088	57.9	51.4	0.139
95% CI	(54.8–61.7)	(49.0–57.9)		(55.1–60.7)	(43.2–59.4)	
Women (%)	32.4	25.2	0.000	33.9	29.3	0.031
95% CI	(30.5–34.3)	(22.4–28.2)		(30.2–37.8)	(27.6–31.1)	
		
HDL						
<40 mg/dl in men (%)	65.1	59.6	0.046	71.8	61.5	0.015
95% CI	(61.6–68.4)	(55.1–63.8)		(63.9–78.6)	(58.7–64.3)	
<50 mg/dl in women (%)	82.9	81.8	0.051	85.8	81.3	0.007
95% CI	(81.3–84.3)	(79.1–84.3)		(82.8–88.4)	(79.7–82.7)	
		
Blood Pressure ≥ 130/85 mm Hg						
Men (%)	32.8	28.8	0.153	30.2	29.1	0.709
95% CI	(28.8–37.1)	(25.7–32.1)		(23.2–38.3)	(26.6–31.9)	
Women (%)	11.6	11.0	0.775	11.7	9.9	0.152
95% CI	(10.4–12.9)	(9.1–13.4)		(10.5–12.8)	(7.7–12.4)	
		
Waist Circumference						
>102 cm in men (%)	18.1	12.2	0.003	17.8	15.2	0.414
95% CI	(15.5–20.9)	(9.6–15.4)		(12.4–25.6)	(13.2–17.4)	
>88 cm in women (%)	48.5	40.6	0.000	57.2	42.6	0.000
95% CI	(46.5–50.6)	(37.3–43.9)		(53.1–61.6)	(40.6–44.4)	
		
Metabolic Syndrome						
Men (%)	29.2	23.4	0.048	32.1	26.5	0.176
95% CI	(26.2–32.4)	(19.9–27.3)		(24.9–40.1)	(24.0–29.1)	
Women (%)	25.5	21.2	0.009	31.3	22.2	0.000
95% CI	(23.5–26.9)	(18.5–24.0)		(27.7–35.1)	(20.6–23.8)	

Prevalences were adjusted by age, calories and alcohol intake, smoking, education, LT and WP physical activity.

Participants of both sexes who reported at least 3 hours of WP activity per day showed a lower prevalence of MS and MS components. For men, 3 hours or more of WP activity significantly improved HDL levels, and for women this activity significantly improved HDL and triglyceride levels, reduced waist circumference, and lowered glucose and MS prevalence (Table 3).

Table 4 reports the adjusted odds ratios and 95% CIs of MS risk across LT and WP physical activity by sex. Metabolic syndrome risk was reduced among men (OR 0.72; 95%CI 0.57–0.95) and women (OR 0.78; 95%CI 0.64–0.94) who were engaged in 30 minutes or more of LT physical activity per day, and among women who reported three hours or more of WP activity a day (OR 0.75; 95%CI 0.59–0.96).

**Table 4:** Adjusted odds ratios of metabolic syndrome by sex, according to leisure-time and workplace activities.

Physical activity	Men	Women	Total
			
Leisure-time	(n = 1484)	(n = 3634)	(n = 5118)
<30 min/day	1	1	1
≥30 min/day	0.72	0.78	0.80
95% CI, p value	(0.57–0.95) 0.047	(0.64–0.94) 0.010	(0.70–0.93) 0.005
			
Workplace	(n = 1005)	(n = 2711)	(n = 3716)
<3 hours/day	1	1	1
≥3 hours/day	0.71	0.75	0.75
95% CI, p value	(0.47–1.09) 0.124	(0.59–0.96) 0.025	(0.61–0.93) 0.011

Odds Ratios were adjusted by age, calories and alcohol intake, smoking, education, LT and WP activity.

## Discussion

This study suggests that at least 30 minutes a day of LT activity at different intensity levels, significantly reduces the risk of MS among men and women. This amount of time is equivalent to a mean energy expenditure of 5.3 METs/day, which corresponds to running 34 minutes per day, at a speed of 10 min/mile [24]. This study also shows that men who engaged in the aforementioned amount and intensity of LT activity, significantly improved their levels of HDL and reduced their waist circumference. Women who spent at least 30 minutes a day in LT activities also significantly improved their triglyceride levels and reduced their waist circumference. These findings are consistent with previous studies demonstrating that regular LT physical activity is an important protective factor against metabolic diseases, because it both prevents and reduces established atherosclerotic risk factors, including elevated triglycerides, low HDL and abnormal waist circumference [10-12,29]. LTPA also reduces type 2 diabetes and MS risk [14-17].

Our results show that engaging in three or more hours a day of WP activities at different intensity levels is associated with a reduction in risk of MS among men and women, although the results for men were not significantly, perhaps due to a lack of statistical power in this group. This amount of time spent in WP activity was associated with a mean energy expenditure of 10.8 METs/day. This amount of energy expenditure is equivalent to walking slowly at 2.5 mph, carrying light objects, or patient care during 216 minutes per day [24]. Our results are consistent with a study that reported that higher occupational PA was found to decrease MS risk in the general adult population of Taiwan [47]. Others studies have also shown that moderate to high levels of occupational activity reduce cardiovascular mortality in both men and women with hypertension and diabetes [12,13,29], and that if occupational activity is high enough, the risk of breast cancer can also be markedly reduced [48].

In Mexico, public health strategies to prevent chronic diseases are based on the MMH recommendations to promote moderate to vigorous PA such as walking, cycling, running, etc. However, this focus may lead to the common misunderstanding that only moderate to vigorous activity improves health [49]. Our results are consistent with prospective and experimental studies indicating that both LT and WP activity at different levels of intensity, including low-intensity activities can also prevent type 2 diabetes [31,32] and MS [32,50]. Based on our results, we suggest that PA recommendations should also emphasize the health benefits of lower-intensity PA. For example, recreational walking, short bouts of activity undertaken at the workplace (such as taking the stairs instead of the elevator), and walking instead of driving shorter distances. Physical activities at home, such as actively playing with children or gardening can also contribute to the amount of daily activity required to improve health [29,51]. However, it is important to point out that lower intensity LT or WP activities may require more total minutes per week to achieve health benefits [50].

Our results do not negate the current recommendations of PA [18-20]. However, based on the fact that our study participants are middle-age, insufficiently active, and that the most common form of LT physical activity in Mexico is walking [52], we consider that it is more feasible to encourage different intensity levels of LT physical activity (including low-intensity) among inactive people, instead of only recommending moderate to vigorous PA [22,32].

Furthermore, LT activities at the workplace should be encouraged among workers who do not receive much exercise in the course of their workday routine. The results of a study conducted in Mexico indicate that at least one third of the workers in a public university reported willingness to engage in LT physical activity at their workplace, and their interest was associated with both higher income and higher levels of education [53]. Studies about the effectiveness of worksite PA promotion and intervention, show that these programs can significantly improve some relevant risk factors (e.g. cholesterol levels, body composition) work-related outcomes (e.g. reduce absenteeism), as well as increase cardio-respiratory fitness and energy expenditure [54-57].

The MMH strategies to increase PA in the Mexican population [58,59] have had little impact on the PA behavior of the population, and on the general health. This failure may be due to a lack of relevant government policy, planning and legislation initiatives to enhance opportunities for PA, poor coordination between the ministries of health, education and sports, and a lack of economic resources for sports infrastructure and institutions [60]. The social side of MS and chronic disease prevention should address people from all economic and educational levels, and account for differences in sex, age and occupation. Guidelines should encourage individuals to change particular aspects of their lifestyle. These changes could be implemented by: 1) developing infrastructure to improve the availability of PA opportunities in sites like parks and sports fields; 2) encouraging active commuting like walking or cycling; 3) proposing more specific public health policies to encourage PA in children, youth, workers, housewives, and elderly people; 4) developing strategies to encourage leisure PA within the family and within the workplace [61]; and 5) investigating the reasons people participate in or avoid PA, so that this information could be used to develop more effective PA promoting strategies.

Some of the limitations of our study include the fact that questionnaire assessments of PA are subject to recall bias [62] and typically overestimate amounts of PA [63,64]. Furthermore, self-reported PA does not provide accurate estimates of absolute amounts of activity (kilocalories per day) [65]. However, these instruments are useful for certain study populations and they have been validated to identify differences in the PA levels of populations [37,38,66,67]. Since our study uses cross-sectional data, it is not possible to fully determine the direction of causality. This is because although poor PA may cause MS, also MS is likely to result in decreased PA. To avoid biases that could modify the association between PA and MS, we eliminated study participants with type 2 diabetes, hypertension and other serious diseases that decrease PA. For example, diabetic individuals tend to not engage in regular PA [68] and hypertensive individuals tend to be more sedentary and partake in less vigorous PA [69].

## Conclusion

Our results indicate that both LT and WP physical activity at different intensity levels, including low-intensity significantly reduce the risk of MS. This finding highlights the need for more recommendations regarding the specific amount and intensity of LT and WP activity needed to prevent MS. We postulate that MS prevention efforts should focus on encouraging individuals to increase their energy expenditure through different types and intensity of PA. Efforts to increase PA should involve multiple groups, including private and government institutions, the health care and education systems, who must join forces to implement programs that incorporate recent evidence regarding the health benefits of physical activity.

## Competing interests

The authors declare that they have no competing interests.

## Authors' contributions

YF, CS, ML and LDDC were involved in drafting the manuscript. EHC, GH, JOT and GCK have given final approval of the version to be published. PMH was responsible for the design of the study, performed the statistical analysis and drafted the manuscript. JS contributed to the study design, coordination and to the original conception and design of the Health Worker Cohort Study. All authors made critical comments during the preparation of the manuscript and fully accept responsibility for the work.

## Pre-publication history

The pre-publication history for this paper can be accessed here:


